# Molecular Characteristics and Antimicrobial Resistance of Group B Streptococcus Strains Causing Invasive Disease in Neonates and Adults

**DOI:** 10.3389/fmicb.2019.00264

**Published:** 2019-02-18

**Authors:** Ming-Horng Tsai, Jen-Fu Hsu, Mei-Yin Lai, Lee-Chung Lin, Shih-Ming Chu, Hsuan-Rong Huang, Ming-Chou Chiang, Ren-Huei Fu, Jang-Jih Lu

**Affiliations:** ^1^Division of Neonatology and Pediatric Hematology/Oncology, Department of Pediatrics, Chang Gung Memorial Hospital, Yunlin, Taiwan; ^2^College of Medicine, Chang Gung University, Taoyuan, Taiwan; ^3^Division of Pediatric Neonatology, Department of Pediatrics, Chang Gung Memorial Hospital, Taoyuan, Taiwan; ^4^Department of Laboratory Medicine, Chang Gung Memorial Hospital at Linkou, Taoyuan, Taiwan; ^5^Department of Medical Biotechnology and Laboratory Science, Chang Gung University, Taoyuan, Taiwan

**Keywords:** group B streptococcus, multilocus sequence typing, antimicrobial resistance, bloodstream infection, invasive disease

## Abstract

We aimed to analyze the molecular characteristics, clonality and antimicrobial resistance profiles of group B streptococcus (GBS) isolates collected in Taiwan from invasive diseases and carriage. Multilocus sequence typing (MLST) was used to assess the genetic diversity of 225 GBS strains from neonates and adults with invasive GBS diseases. 100 GBS strains collected from colonized pregnant women during the same period were compared, and all strains were characterized for one of nine capsule genotypes. We also determined the susceptibilities of all GBS isolates to various antimicrobial agents. The most frequently identified serotypes that caused invasive disease in neonates were III (60.6%) and Ia (17.3%), whereas type VI (32.7%), Ib (19.4%), and V (19.4%) were the most common to cause invasive disease in adults. Serotype VI was the leading type that colonized pregnant women (35.0%). Twenty-six sequence types (STs) were identified, and 90.5% of GBS strains were represented by 6 STs. ST-17 and ST-1 were more prevalent in invasive diseases in neonates and adults, respectively. The majority of serotype III and VI isolates belonged to clonal complex (CC)-17 and CC-1, respectively. ST-17 strains were more likely to cause meningitis and late-onset disease than other strains. In addition, ST-12 and ST-17 GBS strains showed the highest rate of resistance to erythromycin and clindamycin (range: 75.8–100%). In conclusion, CC-17/type III and CC-1/type VI are the most important invasive pathogens in infants and non-pregnant adults in Taiwan, respectively. GBS genotypes vary between different age groups and geographical areas and should be considered during GBS vaccine development.

## Introduction

*Streptococcus agalactiae* (group B *Streptococcus*; GBS) is a commensal flora in human gastrointestinal and genitourinary tracts. However, GBS is the major cause of early-onset sepsis, meningitis, and sometimes late-onset pneumonia in neonates (Joubrel et al., 2015). Colonization in pregnant women and vertical transmission are the major sources of neonatal infection (Lu et al., 2014). In the past decade, GBS has increasingly accounted for invasive disease in non-pregnant adults, including soft tissue infections, bone and joint infections, bacteremia and endocarditis (Crespo-Ortiz Mdel et al., 2014; Björnsdóttir et al., 2016). Recent research has focused on the molecular and epidemiological characteristics of invasive GBS isolates (Kwatra et al., 2015; Medugu et al., 2017; Metcalf et al., 2017), because prevention strategies and development of effective GBS vaccination are still urgently needed in the era of intrapartum antibiotic prophylaxis (Mukhopadhyay et al., 2014; Creti et al., 2017; Li et al., 2017).

Invasive GBS strains are characterized by several virulence factors and various capabilities of host cells invasion, penetration of the blood-brain barrier, and escape from host immune responses, which lead to clinical manifestations (Cutting et al., 2014; Mu et al., 2014; Kwatra et al., 2015). One of the major contributors to virulence is the capsular polysaccharide antigen, which can be used to describe ten different GBS serotypes. Specific GBS serotypes are associated with antibiotic-resistant strains, neonatal diseases, or specific organ involvement (Ferrieri et al., 2013; Alhhazmi et al., 2016). GBS isolates can be further grouped into different sequence types (STs) using multilocus sequence typing (MLST) that identifies sequence variation among conserved housekeeping genes. Some clonal complexes (CCs), defined as genetically related STs that are grouped into clusters following phylogenetic analyses, are associated with multidrug-resistant GBS strains, hypervirulent strains, or clinically important GBS strains (Nagano et al., 2012; Campisi et al., 2016; Martins et al., 2017).

Because the population variability and genetic diversity of GBS isolates could potentially affect the prevention strategy and effectiveness of vaccination campaigns (Bellais et al., 2012), it is important to understand whether specific GBS genotypes are associated with specific clinical manifestations and whether certain GBS genotypes are prevalent in different settings. We investigated the genetic relatedness of invasive GBS strains from neonates and adults using MLST and compared the genotypes identified among pregnant women sampled during the same study period.

## Materials and Methods

From January 2006 to December 2015, 225 GBS strains were isolated from blood and/or cerebrospinal fluid of neonates (*n* = 127) and adult patients (*n* = 98) with invasive GBS diseases. These cases were retrieved retrospectively from the database of Chang Gung Memorial Hospital (CGMH), and all isolates were obtained from the bacterial library of CGMH’s central laboratory. The “colonizing” collection included 100 GBS strains isolated from pregnant women in CGMH during a period that overlapped the study period (2014–2015). Cultures were obtained from women via vaginal-rectal swabs using standard methods as described in previous studies (Kwatra et al., 2016; Medugu et al., 2017). This study was approved by the ethics committee of CGMH, and written informed consents were provided by the pregnant women who had GBS colonization. For patients with GBS invasive diseases, a waiver of informed consent for anonymous data collection was approved.

### Antimicrobial Susceptibility Testing

All GBS isolates were rated for susceptibility to seven antibiotics, including erythromycin, penicillin, clindamycin, vancomycin, ampicillin, cefotaxime, and teicoplanin according to the guidelines of the Clinical and Laboratory Standards Institute for the microdilution minimum inhibitory concentration (MIC) method (Clinical Laboratory Standards Institute, 2014). The double-disk diffusion test was applied to identify inducible clindamycin resistance.

### MLST and Capsule Genotyping

We used MLST to evaluate all GBS isolates and sequenced seven housekeeping genes, as previously described (Manning et al., 2009). Briefly, PCR fragments for seven housekeeping genes (*adhP*, *atr*, *glcK*, *glnA*, *pheS*, *sdhA*, and *tkt*) were amplified and sequenced. The ST was determined via the *Streptococcus agalactiae* MLST database^1^. STs not previously described were submitted to and were assigned by the *S. agalactiae* MLST database. The STs were grouped via the eBURST program into CCs whose members shared at least five of the seven MLST loci (Francisco et al., 2009); otherwise, an ST was considered a singleton.

The capsule genotypes were analyzed using the polymerase chain reaction (PCR) approach, and this assay, as well as the DNA isolation method, was described in our previous publication (Tien et al., 2011).

### Phylogenetic, Epidemiological and Statistical Analysis

A neighbor-joining tree was constructed (Zhang and Sun, 2008) using MEGA5 (Tamura et al., 2011) to build sequence alignments and phylogenetic trees, and this phylogenetic network was applied to 46 parsimonious-informative (PI) sites in SplitsTree4 using the neighbor-net algorithm (Huson and Bryant, 2006). In addition to GBS cluster (CC), recombination between STs was evaluated using the pairwise homoplasy index (PHI).

The frequencies of STs, CCs, and capsular genotypes were assessed by GBS strain sources, and comparisons were made between three different collections (newborn invasive GBS isolates, adult GBS isolates, and pregnant women who had GBS colonization) using the likelihood ratio χ^2^ or Fisher’s exact test. The Mantel-Haenszel χ^2^ test was used to test for trends. Unadjusted odds ratios (ORs) and corresponding 95% confidence intervals (CIs) were calculated, and logistic regression was used to simultaneously identify predictors of infection with specific GBS genotypes. Statistical analyses were performed using SPSS, version 15.0 (IBM SPSS, Chicago, IL, United States).

## Results

Among the 225 invasive GBS strains identified through the 10-year period, 98 were isolated from adult patients, and 127 were isolated from neonates with either early-onset disease (*n* = 34) or late-onset disease (*n* = 93). The majority of neonates (96.1%, 122 patients) were < 3 months old, and 92.1% were term births or late-preterm infants (*n* = 4). A total of 20 cases of meningitis in neonates were identified. In our institute, the incidence of GBS invasive in neonates was 3.4 cases per 10,000 live births (2006–2015). In non-pregnant adult with invasive diseases, 98 GBS isolates were recovered from patients with ages ranging from 15 to 92 years, with a median age of 67.5 years. In all cases of GBS invasive diseases, the primary culture site was blood (*n* = 225), and GBS strains were isolated from the cerebrospinal fluid (CSF) in 20 neonates (data not shown).

### Antibiotic Susceptibility

All of the 325 GBS strains were sensitive to penicillin, ampicillin, and vancomycin. None of these strains displayed cefotaxime resistance. On the other hand, only 48.9 and 51.4% of all GBS strains were sensitive to erythromycin and clindamycin, respectively. Most of the erythromycin resistant GBS isolates (51.1%) were also clindamycin resistant (92.8%, 154 isolates, *p* < 0.001 by Pearson χ^2^ test). As we observed antimicrobial resistance profiles by serotype and ST, we found significantly higher rates of erythromycin and clindamycin resistances were noted in serotypes Ib, III and V. There was no significant difference in the numbers of erythromycin-nonsusceptible or clindamycin-nonsusceptible GBS isolates obtained from different age groups. Erythromycin resistance was found in almost all common CCs, but was especially high among ST12 and ST17.

### Distribution of GBS Capsule Genotypes

Overall, capsular polysaccharide (CPS) type III (34.2%) and VI (22.8%) were the most predominant, followed by type Ia (12.6%), Ib (12.6%), and V (12.6%) (Table 1). The distribution of CPS genotypes by age showed that CPS III (29.4%, *n* = 10) and CPS Ia (29.4%, *n* = 10) were the most common for patients with early-onset disease (EOD). This finding was mirrored for cases of late-onset disease (LOD), for which CPS III (80.0%, *n* = 60) predominated, followed by CPS Ia (12.0%, *n* = 9) (by χ^2^ test, *P* < 0.001). In total, CPS III accounted for 64.2% (70/109) of the strains causing invasive disease in neonates. Few isolates were identified from children aged 91 days to 14.9 years (18/225 cases, 8.0%). Among these cases, CPS III still accounted for 38.9% (7/18 isolates). For colonized GBS strains, types VI (35%) and III (25%) were predominant, followed by types V (16%) and Ib (10%). For GBS strains causing invasive diseases in adults, type VI was predominant (32.6%), followed by types V and Ib (both 19.4%).

**Table 1:** Numbers of invasive GBS strains and colonized GBS strains by capsular polysaccharide capsule (CPS) typing and age.

CPS	No. (%) of cases						
	EOD	LOD	91 days to 14.9 years	15–50 years (invasive)	15–50 years^∗^ (colonized)	>50 years	Total
Ia	10 (29.4)	9 (12.0)	3 (16.7)	6 (26.1)	7 (7.0)	6 (8.0)	41 (12.6)
Ib	7 (20.6)	2 (2.7)	3 (16.7)	5 (21.7)	10 (10.0)	14 (18.7)	41 (12.6)
II	2 (5.9)	1 (1.3)	0 (0)	2 (8.7)	6 (6.0)	5 (6.7)	16 (4.9)
III	10 (29.4)	60 (80.0)	7 (38.9)	2 (8.7)	25 (25.0)	7 (9.3)	111 (34.2)
V	2 (5.9)	2 (2.7)	2 (11.1)	4 (17.4)	16 (16.0)	15 (20.0)	41 (12.6)
VI	3 (8.8)	1 (1.3)	3 (16.7)	4 (17.4)	35 (35.0)	28 (37.3)	74 (22.8)
VII	0 (0)	0 (0)	0 (0)	0 (0)	1 (1.0)	0 (0)	1 (0.3)
Total	34	75	18	23	100	75	325 (100)


*EOD, early-onset disease; LOD, late-onset disease. ^∗^Except for the age group of 15–50 years, all other age groups were invasive GBS strains*.

### Genetic Diversity of GBS

**FIGURE 1:**
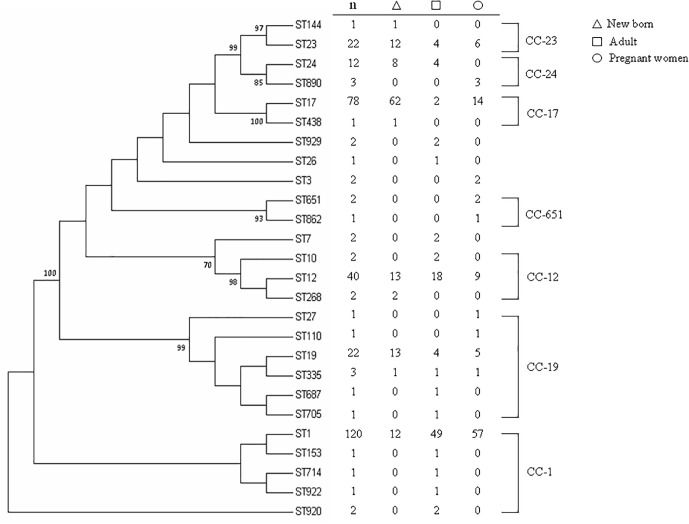
Phylogenetic analysis of MLSTs from 325 GBS strains collected from neonates and adults with invasive disease or colonized pregnant women. The consensus tree was constructed using the neighbor-joining algorithm based on the distance matrix of pair-wise differences between STs. Numbers presented above the branch indicated ST relationships were > 70% bootstrap confidence values based on analysis of 1,000 replicates.

MLST classified the 225 invasive strains into 20 STs and classified the 100 maternal colonizing strains into 12 STs (Figure 1), yielding 26 unique STs. All STs are already published in the MLST database. Seven CCs were classified by bootstrap analysis (Figure 1) and CC-1, CC-19, and CC-12 accounted for the major CCs that contained more than half of the isolates (a total of 198 strains, including 125 strains in CC-1, 44 strains in CC-12, and 29 strains in CC-19). ST-1 was the most prevalent and accounted for 36.9% of all strains in this epidemiological survey. Neighbored STs in CC-23, CC-24, and CC-17 had greater than or equal to 70% bootstrap confidence value and thus indicated higher genomic diversity among them. In contrast, most of the STs in CC-1, CC-19, and CC-12 were identified with < 70% confidence value, which implied a higher similarity of genome composition among these STs (Figure 1).

**FIGURE 2:**
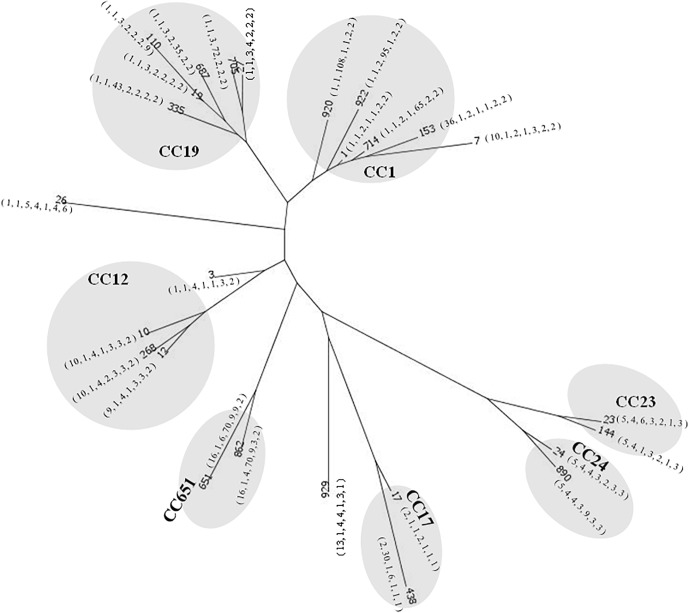
Phylogenetic comparison of a total of 3,457 nucleotides using the neighbor-net algorithm for 325 strains of GBS representing 26 STs. Gray circles represent STs in the same clone complex (CC). The number labeled in quotation marks under each ST represent its MLST profile, which is ordered by the locus of *adhP, pheS, glnA, sdhA, glcK*, and *tkt*.

With regard to the source of these isolates, most of the invasive strains in adults and the colonized strains in pregnant women were ST-1, whereas ST-17 was the most predominant strain in newborns with invasive disease (Figure 1). The phylogenetic network was generated in order to compare the diversity of each ST and is presented in Figure 2. Most of the neighbored STs in CC-1, CC-19 and CC-12 showed only locus variations between each other ST (Figure 2) and indicated their genome diversity was closely related, because lower bootstrap confidence was also noted in these CCs, as shown in Figure 1. The number in Figure 1 phylogenetic tree represents percentage of assessments of confidence that two CC groups contain sequence variation between each other. Based on our bootstrap analysis (Figure 1), CC-651 and CC-17 contain high value confidence, which means higher sequence variation between ST-17, ST-438 in CC-17 and ST-651, ST-862 in CC-651. Furthermore, a single nucleotide polymorphism identified in *atr* led to the single locus variation identified in CC-1, CC-19 and CC-23 and as a result, made ST-920, ST-27 and ST-144 far away from other STs in these CCs. Although other single locus variations, such as *glhA*, *sdhA*, and *tkt* were also identified in the above CCs, none of them appear to have caused greater genomic diversities among each of the STs (Figure 2).

**FIGURE 3:**
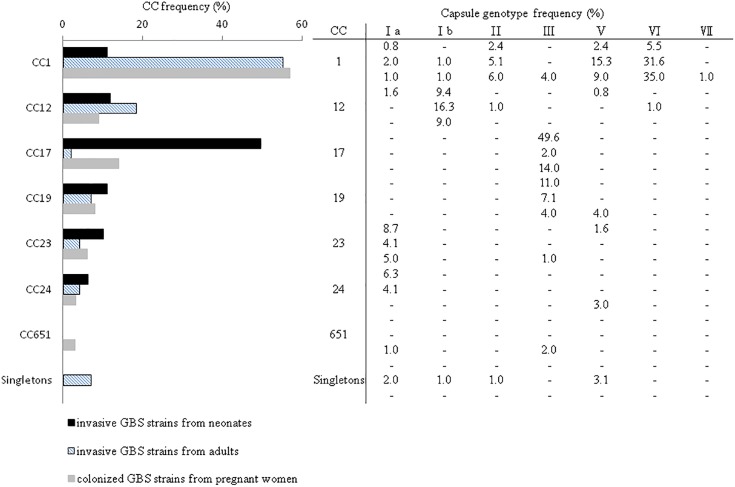
Distribution of GBS CCs as determined by MLST and frequency of cps genotypes among all 325 GBS strains from neonates with invasive disease (*n* = 127), adults with invasive disease (*n* = 98), and women colonized during pregnancy (*n* = 100). Singletons refer to the STs that were not associated with a CC or GBS cluster.

Six STs were common to both colonizing and invasive specimens (Table 2), whereas 13 and 6 STs were unique to invasive and colonizing strains, respectively (Figure 1). Six of the 11 common STs accounted for 90.5% of strains (Table 2), and 4 STs occurred in various frequencies between invasive and colonizing strains. The most notable difference was observed in the frequency of ST-17, which was significantly more common in neonatal invasive strains (OR, 5.18, 95% CI, 1.55–14.21; *P* < 0.001). In contrast, ST-1 (OR, 3.22, 95% CI, 1.23–7.12; *P* < 0.001) predominated in colonizing strains and invasive strains of adults versus neonatal strains. These epidemiological analyses (Figure 3) were based on CCs as the major groups which rely on the similar frequency differences identified between the STs and collections.

**Table 2:** Distribution of the predominant GBS MLSTs from neonatal and adult invasive strains and maternal colonizing strains from Taiwan, 2006–2015.

ST (no. of strains)	No. (%) of strains			
	Overall (total *n* = 325)	Invasive (neonates) total *n* = 127	Invasive (adults) total *n* = 98	Colonizing, total *n* = 100
ST1	120 (36.9)	14 (11.0)	49 (50.0)	57 (57.0)
ST12	40 (12.3)	13 (10.2)	18 (18.4)	9 (9.0)
ST17	78 (24.0)	62 (48.8)	2 (2.0)	14 (14.0)
ST19	22 (6.8)	13 (10.2)	4 (4.1)	5 (5.0)
ST23	22 (6.8)	12 (9.4)	4 (4.1)	6 (6.0)
ST24	12 (3.7)	8 (6.3)	4 (4.1)	0 (0)
Others	31 (9.5)	5 (3.9)	17 (17.3)	9 (9.0)


**FIGURE 4:**
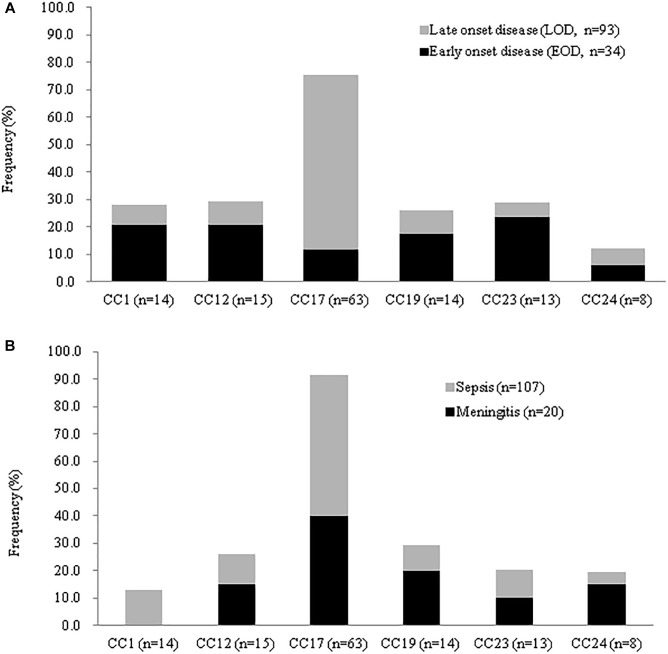
Frequency of disease stratified by GBS CCs among invasive strains recovered neonates in CGMH. **(A)** Percentage of EOD and LOD cases by CC isolated from 127 neonates with invasive disease. **(B)** Disease severity by CC for 127 neonates with neonatal disease and GBS isolation from either blood or CSF.

CC-17 strains were significantly more common in neonates with both EOD and LOD relative to invasive disease in adult or pregnant women (Figure 4), whereas CC-1 strains were more likely to cause invasive diseases in adults and colonization in pregnant women. LOD was most common in neonates infected with CC-17 strains (Table 2); however, CC-17 strains were much more likely than other strains to cause LOD than EOD. CC-17 was found only as CPS III and accounted for 80.5% of all type III GBS in neonatal invasive diseases and 70.3% of all type III GBS strains in our cohort (Figure 4). Furthermore, CC-17 and CC-19 isolates were responsible for 40.0 and 20.0% of neonatal meningitis, respectively (Figure 4).

## Discussion

This study combined genetic, molecular, epidemiological and antimicrobial resistance analyses of the circulating GBS isolates in Taiwan and thereby contributed to more complete information regarding the predominating GBS genotypes among colonized mothers and in patients with invasive disease in Taiwan. We found that CC-17 strains caused more neonatal invasive diseases than other GBS lineages and were responsible for most meningitis cases. CPS type III remains the most predominant serotype of EOD as well as LOD, and is almost exclusively associated with CC-17. These data are very similar to those described in previous studies (Edmond et al., 2012; Bekker et al., 2014; Clinical Laboratory Standards Institute, 2014; Morozumi et al., 2014; Joubrel et al., 2015). In adults, the most commonly identified serotypes among the colonizing isolates were types VI, III, and V, whereas types VI, Ib and V were the predominant strains associated with invasive diseases. These findings have significant impact on serotype-based vaccine development because persistent serotype-specific antibody concentration after vaccination can provide protection more efficiently against EOD or LOD of the locally prevalent strain.

Clinidamycin and erythromycin are the options for treating patients who are allergic to penicillin, and vancomycin can be given in cases of clindamycin- and erythromycin-resistant GBS infection. We found that certain serotypes and CCs were significantly associated with antibiotic resistance to erythromycin or clindamycin, and significant differences were found in the resistance rates among the serotypes in terms of invasive neonatal strains and colonized maternal strains. These results are different from those of previous reports, which found the antibiotic resistance was not correlated with any specific ST or serotype (Wang et al., 2013; Morozumi et al., 2014; Guan et al., 2018). Previous studies in Taiwan found that GBS resistance to macrolide antibiotics is much higher (58.3%) than western countries, and ranges from 11.5 to 32% (Wang et al., 2010, 2013; Martins et al., 2012, 2017). Furthermore, in accordance with a previous report that found a correlation between serotype Ib GBS strain and macrolide and clindamycin resistance (Wang et al., 2010), we found increasing antibiotic resistance among type III and V GBS isolates, and particularly CC-12 and CC-17 strains. The continuing emergence of multi-drug resistant GBS isolates will limit antibiotic treatment options. Because detailed information of GBS sequence or strain type can be practical and important for new vaccine development, further study of resistance genes and molecular characterization of macrolide and tetracycline resistant GBS strains in Taiwan is needed.

GBS isolates colonized in pregnant women are considered to be the reservoirs of invasive strains in neonates. Therefore, previous studies found colonizing GBS isolates had serotype distributions that closely mirrored those of invasive neonatal GBS diseases in the same geographic area (Berardi et al., 2013; Medugu et al., 2017; Teatero et al., 2017). However, the genetic diversity is relatively higher than we previously thought. In our cohort, we found that the molecular characteristics of colonized GBS strains in pregnant women and invasive neonatal strains were quite different, and the switching of molecular characteristics has been found during vertical transmission (Crespo-Ortiz Mdel et al., 2014; Björnsdóttir et al., 2016; Li et al., 2018). Some colonizing genotypes possibly are better adapted for transmission to susceptible neonates and may comprise certain CCs. These strains are less likely to be eradicated by intrapartum antibiotic prophylaxis (Medugu et al., 2017). Furthermore, the transmission routes or sources of LOD GBS infection remains poorly understood, and we also found type III/CC-17 strains were more common in LOD than EOD (Clinical Laboratory Standards Institute, 2014).

In our cohort, we found serotype VI GBS strain was predominant in invasive GBS diseases in adults and colonization in pregnant women; these findings were in contrast with previous studies that found that serotypes III, Ia and V were the predominant GBS strains in adults (Clinical Laboratory Standards Institute, 2014; Medugu et al., 2017; Teatero et al., 2017; Li et al., 2018). Serotype VI GBS was reported to be more common in Asia (Russell et al., 2017), although most studies found it accounted for less than 20% of adult cases and was even rare in neonatal invasive diseases or colonization (Islam et al., 2016; Madrid et al., 2017; Russell et al., 2017). The serotype VI, ST-1 GBS clone has emerged in Taiwan (Wang et al., 2016; Wu et al., 2017), and recent reports suggested that this strain is sexually transmitted and is better adapted to sexual transmission (Wang et al., 2016; Wu et al., 2017). Furthermore, serotype VI/ST1 strains are also common in Japan (Morozumi et al., 2016) and have been reported sporadically in Europe, North America, and Australia during the past decade (Manning et al., 2003; Tsolia et al., 2003; Zhao et al., 2008). Although colonized serotype VI/ST-1 GBS strains were reported to have reduced susceptibility to penicillin in Japanese patients (Kimura et al., 2011), this penicillin resistant VI/ST-1 GBS strain was not observed in the invasive pathogen in Taiwan (Wang et al., 2014) and in our cohort. These findings highlight the significant variation of invasive GBS serotypes among geographic regions and age groups; therefore, specific serotypes should be considered when public health officials develop glycoconjugated GBS vaccines for patients in different areas.

A series of studies have defined type III/CC-17 GBS as hypervirulent due to its epidemiological relevance; this lineage account for the majority of neonatal LOD and meningitis cases (Clinical Laboratory Standards Institute, 2014; Joubrel et al., 2015; Alhhazmi et al., 2016; Campisi et al., 2016; Creti et al., 2017). These studies found that the CC-17 lineage was capable of capsular switching due to recombination of the entire *cps* locus (Bellais et al., 2012) and of becoming multi-drug resistant (Bellais et al., 2012; Campisi et al., 2016; Martins et al., 2017). Manning et al. (Clinical Laboratory Standards Institute, 2014) found that CC-17 strains were more likely to cause meningitis than all other genotypes, even though type III GBS with other lineages (e.g., CC-19) were less likely to be associated with meningitis (Clinical Laboratory Standards Institute, 2014; Joubrel et al., 2015). This can be explained by the presence of surface protein HvgA, a factor that is normally expressed by CC-17 strains and is associated with an increased ability to cross the blood-brain barrier (Bellais et al., 2012; Tazi et al., 2012). However, other virulence genes may also contribute to its invasive and adaptive characteristics. Thus, further research regarding the genetic variation between GBS clonal groups using whole-genome sequencing is warranted.

This study has some limitations. All GBS strains were obtained at a single center in north Taiwan, and further research enrolling multiple medical centers is indicated in order to obtain a more representative view of the genetic characteristics in Southeast Asia. The colonized GBS isolates were obtained between 2014 and 2015, and thus we can not identify the trends in GBS colonization in pregnant women in 2006 versus the trends in 2015. Further, the invasive GBS strains were based on historical data, and we can not rule out the possibility of underreporting or false-negative cases during such a prolonged study period. Finally, the emergence of more virulent GBS types was not documented in this study.

## Conclusion

We assessed the genetic diversity of GBS isolates from neonates and adults with invasive disease and colonized pregnant women, which provide the genetic backbone for development of GBS vaccination in Taiwan. CC-17/type III and CC-1/type VI strains commonly caused neonatal diseases and invasive diseases in adults, respectively. In addition, because ST-17 strain was the most common cause of LOD and meningitis in Taiwan, further genetic studies regarding the transmission, regulation and virulence are needed. In the near future, additional GBS strains should be collected from areas throughout Taiwan and further epidemiological data should be analyzed in order to provide a basis for infection control.

## Author Contributions

M-HT conceptualized and designed the study, drafted the initial manuscript, and approved the final manuscript as submitted. J-FH designed the data collection instruments, and coordinated and supervised data collection and the whole study. L-CL helped to perform the statistical analysis of this study and interpretation of MLST analysis. M-YL helped to collect and verify the data. S-MC performed the microbiological characteristics of this study. H-RH took care of these patients, and carried out the initial analyses. M-CC took care of these patients, and helped data verification. R-HF took care of these patients, and helped data verification. J-JL critically reviewed the manuscript, revised the manuscript, and approved the final manuscript as submitted.

## Conflict of Interest Statement

The authors declare that the research was conducted in the absence of any commercial or financial relationships that could be construed as a potential conflict of interest.

## Footnotes

1 https://pubmlst.org/sagalactiae/
